# Hydrophobicity of Residue 128 of the Stress-Inducible Sigma Factor RpoS Is Critical for Its Activity

**DOI:** 10.3389/fmicb.2017.00656

**Published:** 2017-04-26

**Authors:** Tadayuki Iwase, Takashi Matsuo, Saiko Nishioka, Akiko Tajima, Yoshimitsu Mizunoe

**Affiliations:** ^1^Department of Bacteriology, The Jikei University School of MedicineTokyo, Japan; ^2^Graduate School of Materials Science, Nara Institute of Science and TechnologyNara, Japan

**Keywords:** RpoS, missense mutation, stress response, Shiga toxin-producing *E. coli* (STEC), clinical isolates, food-borne pathogens

## Abstract

RpoS is a key stress-inducible sigma factor that regulates stress resistance genes in *Escherichia coli*, such as the *katE* gene encoding catalase HPII and the *glg* genes encoding glycogen synthesis proteins. Monitoring RpoS activity can provide information on the stress sensitivity of *E. coli* isolates in clinical settings because the RpoS in these isolates is often mutated. In the present study, we found a novel, missense point mutation at RpoS residue 128 in a clinical Shiga toxin-producing *E. coli* (STEC) isolate. This mutation caused RpoS dysfunction and increased stress sensitivity. A mutant *rpoS* was cloned from a clinical STEC that is vulnerable to cold temperature and oxidative stresses. Mutant RpoS protein expression was detected in the clinical isolate, and this RpoS was non-functional according to HPII activity and glycogen levels, which are positively regulated by RpoS and thus are used as indicators for RpoS function. A reporter assay with β-galactosidase indicated that the dysfunction occurred at the transcriptional level of genes regulated by RpoS. Furthermore, substitution analysis indicated that the hydrophobicity of the amino acid at residue 128 was critical for RpoS activity; the simulation analysis indicated that the amino acids of RNA polymerase (RNAP) that interact with RpoS residue 128 are hydrophobic, suggesting that this hydrophobic interaction is critical for RpoS activity. In addition, substitution of Ile128 to Pro128 abolished RpoS activity, possibly as a result of disruption of the secondsary structure around residue 128, indicating that the structure is also a crucial factor for RpoS activity. These results indicate that only one point mutation at a hydrophobic residue of the complex formed during transcription leads to a critical change in RpoS regulation. Moreover, we found that Ile128 is widely conserved among various bacteria: several bacterial strains have Met128 or Leu128, which are hydrophobic residues, and these strains had similar or higher RpoS activity than that observed with Ile128 in this study. These data indicate that the hydrophobicity of the amino acid at residue 128 is critical for RpoS activity and is consequently important for bacterial survival. Taken together, these findings may contribute to a deeper understanding of protein functional mechanisms and bacterial stress responses.

## Introduction

Organisms have stress response mechanisms to protect themselves from environmental stresses (Feder and Hofmann, 1999; Cabiscol et al., 2000). Shiga toxin-producing *Escherichia coli* (STEC) are found in the guts of cattle and they can survive under severe environmental stress conditions, including those in soil, river, and ground water, and they can infect humans (Rasmussen and Casey, 2001; Muniesa et al., 2006; van Elsas et al., 2011; van Overbeek et al., 2014). A greater understanding of the bacterial stress response can provide information for better control of bacterial infections.

RpoS is a key stress-inducible sigma factor (Hengge-Aronis, 1993; Klauck et al., 2007; Dong and Schellhorn, 2010; Battesti et al., 2011; Landini et al., 2014) that regulates stress resistance genes such as the *katE* gene encoding catalase HPII and the *glg* genes encoding glycogen synthesis proteins (Weichart et al., 1993; Tanaka et al., 1997) by binding RNA polymerase (RNAP) and the 5′ upstream region of the genes in *E. coli* (Hengge-Aronis, 2002; Mooney et al., 2005; Typas and Hengge, 2006; Typas et al., 2007). Recently, X-ray crystallographic analysis for the transcription initiation stage was reported, where the binding mechanism among RpoS, RNAP, and oligonucleotides was disclosed (Liu et al., 2016).

Mutated RpoS is often present in clinically isolated *E. coli* strains (Notley-McRobb et al., 2002; Dong et al., 2009), and strains with non-functional RpoS proteins are generally sensitive to stresses (Hengge-Aronis, 1993; Landini et al., 2014). However, RpoS dysfunction may be advantageous under certain conditions, such as those with scarcity of carbon sources (Ferenci, 2008; Chiang et al., 2011). The *rpoS* gene is considered as polymorphic (Jordan et al., 1999; Notley-McRobb et al., 2002; Martinez-Garcia et al., 2003), which influences the trade-off between self preservation and nutritional competence (SPANC; Ferenci, 2003; Ferenci and Spira, 2007). The phenotypic diversity observed in clinical isolates is at least partially attributable to diverse RpoS levels among isolates and the effect of these RpoS levels on SPANC (Levert et al., 2010). Because the presence of scarce carbon sources, readily selects for the loss of RpoS function in both laboratory (Chen et al., 2004) and pathogenic strains (Dong et al., 2009), stressful environmental conditions, such as scarce carbon and nutrient sources, may select for RpoS mutants in environmental *E. coli* populations. Once the RpoS protein is mutated, mutant RpoS is promptly degraded by proteinase owing to the strict regulation of the cellular RpoS level (Zhou and Gottesman, 1998; Becker et al., 2000; Klauck et al., 2001; Hengge, 2009; Battesti et al., 2015).

While surveying RpoS from clinical isolates to investigate the stress tolerance of these pathogens, we identified an STEC clinical strain (Kai1), isolated from a patient with STEC infection in Japan, that is highly sensitive to H_2_O_2_ oxidative stress but nonetheless expresses RpoS. In the present study, we cloned *rpoS* and sequenced it to identify the mutations, and investigated the mechanisms underlying RpoS dysfunction. As a result, we found that hydrophobicity and secondary structure preservation at Ile128 determine RpoS dysfunction.

## Materials and methods

### Bacterial strains and culture conditions

The *E. coli* strain Kai1 is a clinical isolate in the strain collection of the Jikei University School of Medicine in Japan. The laboratory *E. coli* strains K−12 W32110 and K−12Δ*rpoS* were also employed in this study. In addition, K−12Δ*rpoS* with *rpoS* variants having amino acid substitutions, e.g., Ala, Arg, Asn, Asp, Gln, Glu, Leu, Lys, Met, Phe, and Pro, at residue 128 were generated. *E. coli* K−12 DH5α was used as a host for genetic manipulation. Strains harboring an *osmY* promoter-*lacZ* transcriptional fusion on a single copy λ-prophage and strains with a deficiency in RpoS were created by P1 transduction (Banta et al., 2013); these strains were a gift from Prof. Gourse. The bacterial strains used in this study are listed in Table S7.

Bacteria (10^6^ CFU/ml) were inoculated into LB broth (BD Biosciences, USA) and cultured at 37°C; all experiments were carried out on stationary phase cultures (16 h of growth), except for the glycogen assay and reporter assay.

### Cloning of *rpoS* with its intact promoter

The *rpoS* sequence, which included its intact promoter (Takayanagi et al., 1994) was amplified by PCR with forward (5′-ACGAATTCTTAACATGGGTAGCACCGGAA-3′) and reverse (5′-GGAAGCTTTTACTCGCGGAACAGCG-3′) primers. The forward primer had an *Eco*RI recognition site at the 5′ end of the oligonucleotide, and the reverse primer had a *Hin*dIII recognition site at the 5′ end of the oligonucleotide. The amplicon and pSTV28, a low-copy-number plasmid with the chloramphenicol-resistant gene (Takara, Japan) and 15A origin as derived from p15A, were digested by *Eco*RI and *Hin*dIII and then ligated. The plasmid harboring *rpoS* was introduced in strain DH5α and then transferred into K−12Δ*rpoS*.

### Generation of *rpoS* variants with point mutations

To generate *rpoS* variants, *rpoS*^K−12^ was used as a template, and point mutations were introduced using a PrimeSTAR Mutagenesis Basal Kit (Takara). The *rpoS* variants were introduced in strain DH5α and then transferred to K−12Δ*rpoS*. Primers used in this study are listed in Tables S8.

### Measurement of catalase HPII activity

Bacteria were cultured on LB agar for 16 h. Twenty micrograms (wet weight) of bacterial cells were suspended in 100 μl of saline solution. Bacterial suspensions were heated at 55°C for 15 min. One hundred microliters of 1% Triton X-100 (Sigma) and 30% H_2_O_2_ solution was added to a test tube containing 1000 μl of bacterial suspensions, and catalase activity was measured as the height of the foam that formed (Iwase et al., 2013).

### Glycogen assay

Bacteria were cultured for 24 h at 37°C on Kornberg agar (1.1% K_2_HPO_4_, 0.85% KH_2_PO_4_, 0.6% yeast extract, 1.5% agar, 1% glucose, and 1.5% agar; Govons et al., 1969; Liu and Romeo, 1997; Wei et al., 2000; Iwase, under review). Ten micrograms of wet weight bacterial cells were suspended in 100 μl of saline solution and heated at 95°C for 15 min for enzyme denaturation. Following heat treatment, the samples were sonicated to disrupt the bacterial cells and then centrifuged at 10,000 *g* at 4°C for 30 min to remove bacterial debris. The resulting supernatants were further filtrated to obtain clarified samples. Three microliters of 2% iodine solution (2% iodine/1 M NaOH, Wako Pure Chemical Industries, Japan) were added to the filtrated supernatants (100 μl) and colorization was then measured spectrophotometrically within 5 min (492 nm).

### Reporter assay

β-Galactosidase assays were performed for evaluating RpoS activity using a strain harboring an *osmY* promoter-*lacZ* transcriptional fusion on a single copy λ-prophage and a strain with a deficiency in RpoS (Banta et al., 2013). Bacteria were cultured in LB broth with 2-nitrophenyl-β-D-galactopyranoside (ONPG; Thermo Fisher) at 37°C for 6 h, and color changes were measured using a spectrophotometer at 492 nm. Bacteria were also cultured in LB broth with X-gal (Sigma-Aldrich), which was used for qualitative analysis. In the presence of β-galactosidase activity, bacterial cells were stained blue.

### mRNA expression analysis

mRNA expression in bacteria harvested at O/N culture (16 h) was analyzed using quantitative reverse transcriptional PCR (qRT-PCR) with gene-specific primers and SuperScript III Platinum One-Step qRT-PCR Kit (Invitrogen), according to manufacturer instructions. *rrsA* encoding 16S rRNA was used as a reference gene for normalization of qRT-PCR. Primers used in this experiment are shown in Table S9.

### Oxidative stress test

Bacteria were cultured in LB broth at 37°C for 16 h. In the oxidative stress test, 20 mM H_2_O_2_ aqueous solutions were used as previously described with some modifications (Wang et al., 2011). Bacterial culture (10 μl) was added to 990 μl of 20 mM H_2_O_2_ aqueous solutions and was incubated at 25°C for 2 h. Fifty microliters of the suspension was plated onto LB agar and cultured at 37°C for 16 h for enumeration of bacterial counts to determine survival after H_2_O_2_ exposure.

### Cold stress test

Bacteria were cultured in LB agar at 37°C for 16 h and incubated at 4°C during the study period. Cold-stressed bacteria on LB agar were cultured in LB broth at 37°C for 24–48 h for enumeration of bacterial counts to determine survival after cold- stress exposure.

### Molecular dynamics (MD) calculation

MD calculations were conducted on the YASARA structure molecular modeling software package (Ver.14.4.15; Krieger et al., 2002). The calculations were run using AMBER 03 force field, the aqueous solution model with 0.9% NaClaq ion concentration, point charges assigned at *pH* = 7.4 and additional Na^+^ or Cl^−^ for charge neutralization in a cubic cell boundary defined at 5 Å from the protein surface. Simulations were started from the structural data of fragment Leu114-Asp135 in RpoS taken from the previously reported structure of RpoS-RNAP-oligonulectotide complex (PDB: 5IPL) and conducted for the solution model at 310.15 K. Calculations were continued over 12 ns until Cα-RMSDs (root-mean-square deviations) reached at equilibrium. Snapshot figures every 25 psec were stored.

### Conservation of amino acid at residue 128 of RpoS among various bacteria

The *rpoS* sequences of gram-negative bacteria were randomly selected in the NCBI gene database and were analyzed by the ClustalW, a multiple sequence alignment program, (DNA Data Bank of Japan).

### Statistical analysis

For multiple group comparisons, analysis of variance (ANOVA) was performed, and the significance of differences was evaluated using the Scheffe's F test if the ANOVA results were significant. A value of *P* < 0.05 was considered to indicate statistical significance. Calculations were performed using Excel software (Microsoft, US) and Statcel (OMS, Japan).

## Results

### Sequence analysis of the *rpoS* gene in strain Kai1

To investigate the function of RpoS in strain Kai1, we compared the sequence of the Kai1 *rpoS* gene (*rpoS*^Kai1^) with that of strain K−12 (*rpoS*^K−12^), encoding a functional RpoS. We found two missense point mutations in *rpoS*^Kai1^ compared to *rpoS*^K−12^: an amino acid substitution at residue 33 (Gln to Glu) and one at residue 128 (Ile to Asn; Table 1). The substitution of residue 33 is often observed in clinical isolates with functional RpoS proteins (Atlung et al., 2002; Subbarayan and Sarkar, 2004). The substitution of residue 128, however, has not been previously reported. Therefore, it was possible that the RpoS dysfunction was due to the substitution of residue 128.

**Table 1:** **Missense point mutations in *rpoS* in Kai1 strain**.

	**Residue 33**	**Residue 128**
*rpoS*^K−12^	CAG (Gln)	ATC (Ile)
*rpoS*^Kai1^	GAG (Glu)	AAC (Asn)

### *rpoS* with substitutions at residues 33, 128, or 33 and 128 expressed RpoS protein

**Figure 1:**
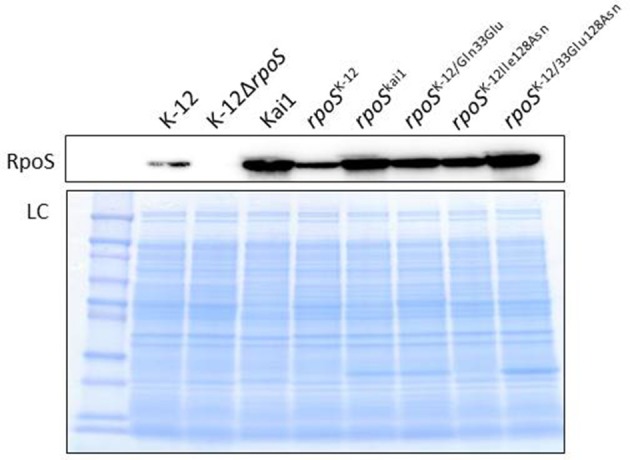
**Effect of substitution at residue 128 on RpoS expression**. RpoS expression in *rpoS* variants harboring mutation(s) in *rpoS*^Kai1^ (*rpoS*^K−12/Gln33Glu^, *rpoS*^K−12/Ile128Asn^, and *rpoS*^K−12/Glu33Asn128^) and in the Kai1 strain. The experiment was conducted thrice, and representative images are shown. LC, loading control. The loading control image depicts a part of Coomassie stains of SDS-PAGE gels.

To determine whether the substitution of residues 33 or 128 affected RpoS protein expression, we first cloned *rpoS*^Kai1^ and *rpoS*^K−12^ into a low-copy plasmid pSTV28 and generated plasmids harboring *rpoS*^K−12^ with the mutation(s) found in *rpoS*^Kai1^ (*rpoS*^K−12/Gln33Glu^, *rpoS*^K−12/Ile128Asn^, and *rpoS*^K−12/Glu33Asn128^). Subsequently, we introduced them into the K-12Δ*rpoS* strain and then performed western blotting for RpoS protein. All *rpoS* variants expressed RpoS (Figure 1). The data showed that *rpoS* with mutations observed in *rpoS*^Kai1^ still expressed the RpoS protein (Figure 1), consistent with RpoS expression observed in strain Kai1 (Figure 1).

### RpoS activity is affected by the residue 128 substitution

**Figure 2:**
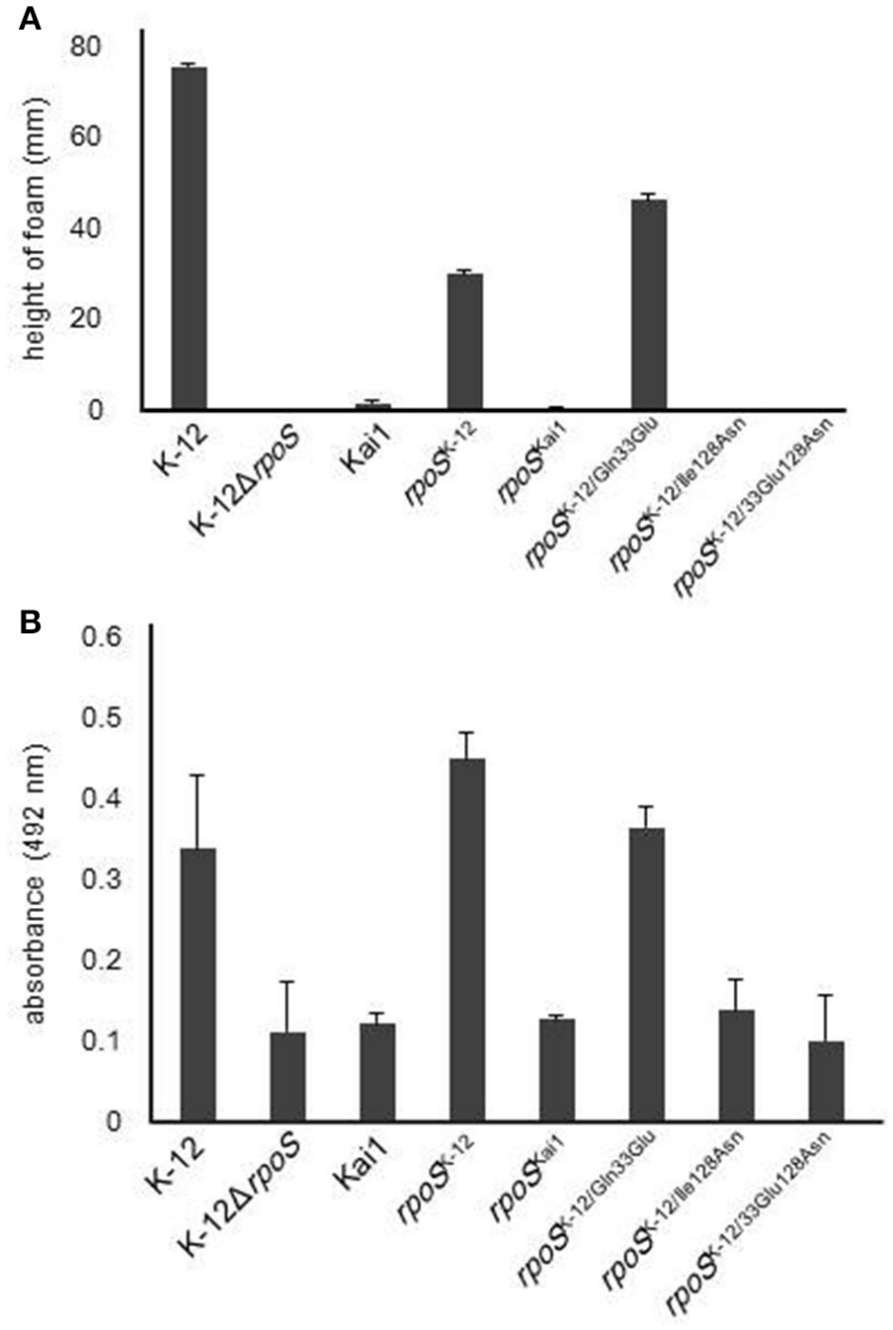
**Effect of substitution at residue 128 on RpoS activity**. RpoS activity in *rpoS* variants was determined by HPII activity assays and analysis of glycogen levels, which are positively regulated by RpoS. **(A)** HPII activity in *rpoS* variants was measured by HPII assays. **(B)** Glycogen levels in *rpoS* variants were measured by glycogen assays. Mean values are shown (*n* = 3). Error bars represent standard deviations. The statistical significance of the differences is tabulated in Tables S1, S2.

Next, to investigate the effects of the substitution of residues 33 or 128 on the RpoS activity, we measured HPII activity and glycogen levels, which are positively regulated by RpoS and used as an indicator for RpoS activity (Weichart et al., 1993; Iwase et al., 2013). Both were low or undetectable in the *rpoS* variants containing the substitution at residue 128, while the substitution of residue 33 did not affect HPII activity (Figure 2A) or glycogen levels (Figure 2B).

### RpoS dysfunction via the residue 128 substitution occurs at the transcriptional level of the RpoS-regulated genes

**Figure 3:**
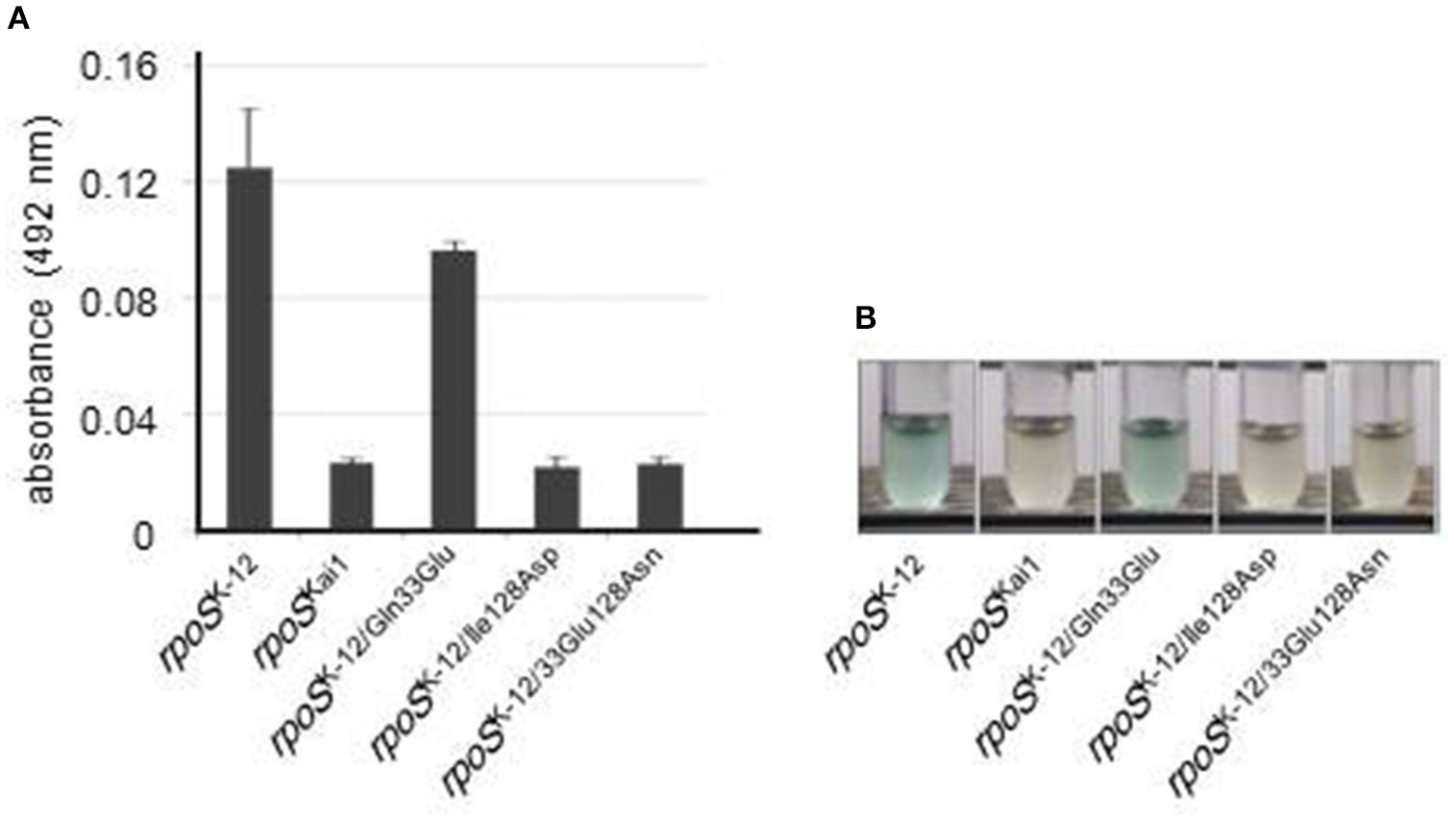
**RpoS dysfunction via substitution at residue 128**. Reporter assays with β-galactosidase were performed for evaluation of RpoS function using a strain in which intact *rpoS* was deleted and an *osmY* promoter-*lacZ* transcriptional fusion was located on a single copy λ-prophage and using each *rpoS* variant. **(A)** β-Galactosidase activity was measured in the presence of ONPG, a substrate of β-galactosidase, using spectrophotometry. Mean values are shown (*n* = 3). Error bars represent standard deviations. **(B)** β-Galactosidase activity was visualized using X-gal, a substrate of β-galactosidase that produces the insoluble blue dye indigo. The experiment was conducted thrice, and representative images are shown. The statistical significance of the differences is tabulated in Table S3.

To investigate whether the residue 128 substitution affected in gene expression of RpoS-regulated genes, a reporter assay using β-galactosidase activity were performed. In the strains harboring *rpoS* with the residue 128 substitution, low β-galactosidase activity was observed (Figure 3). This result shows that the RpoS dysfunction resulted from the residue 128 substitution.

Taken together, the RpoS dysfunction observed in strain Kai1 is due to the substitution of residue 128 (Ile to Asn) in *rpoS*^Kai1^.

### RpoS activity is affected by the hydrophobicity of residue 128

**Figure 4:**
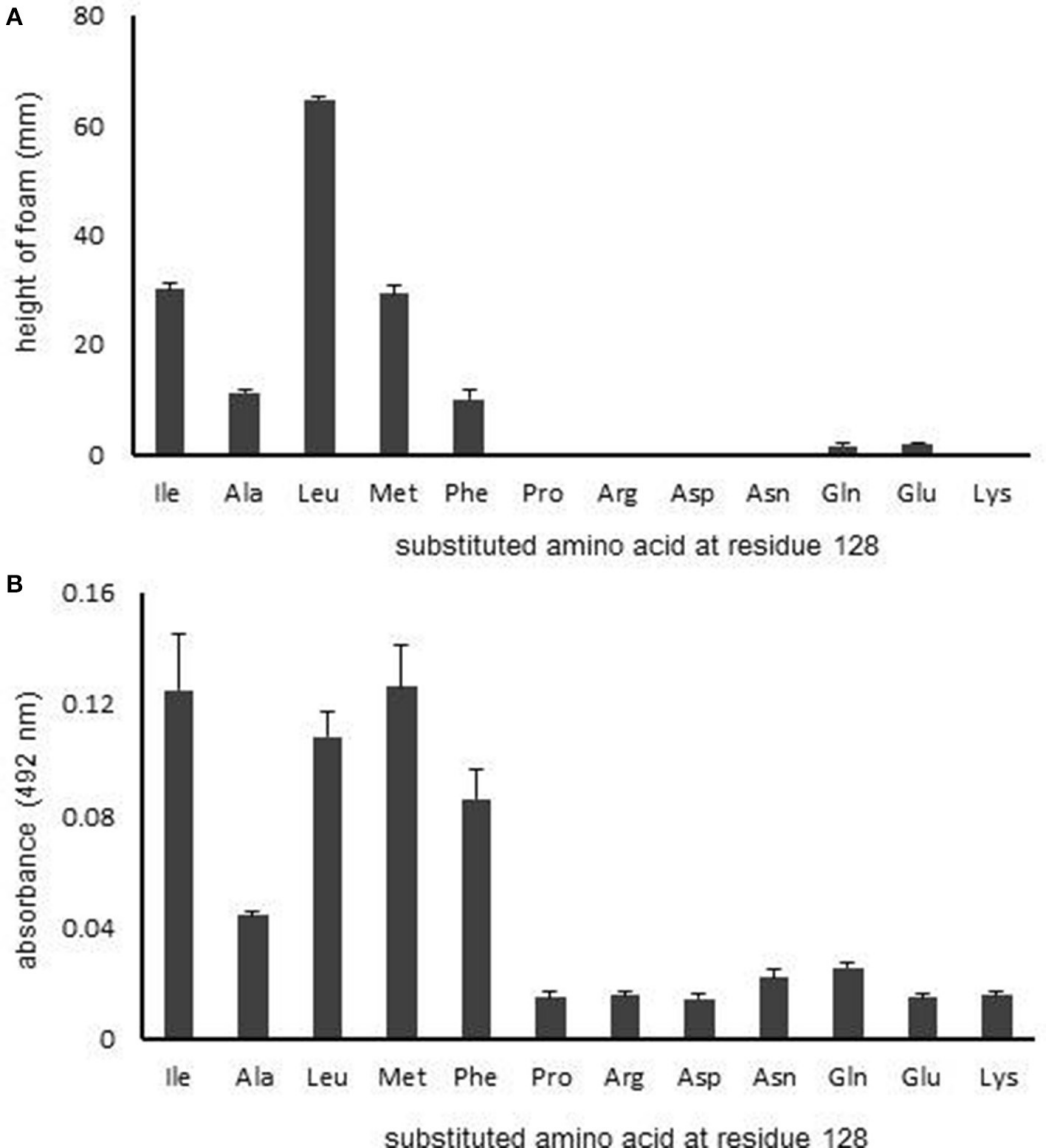
**Effect of the characteristics of the amino acid at residue 128 on RpoS activity. (A)** HPII activity in *rpoS* variants was measured by HPII assays. **(B)** β-Galactosidase activity was measured using spectrophotometry. Mean values are shown (*n* = 3). Error bars represent standard deviations. The statistical significance of the differences is tabulated in Tables S4, S5.

Next, to further investigate the significance of residue 128 on RpoS activity, we evaluated the effect of various amino acid substitutions at this position on RpoS activity using the HPII assay (Figure 4). The following amino acids were investigated: positively charged and hydrophilic amino acids (Arg and Lys), negatively charged and hydrophilic amino acids (Asp and Glu), an electrostatically neutral and hydrophilic amino acid (Gln), hydrophobic amino acids (Ala and Pro), a hydrophobic branched-chain amino acid (Leu), a hydrophobic amino acid containing sulfur (Met), and a hydrophobic amino acid containing an aromatic ring (Phe).

The *rpoS* variants that contained a hydrophobic amino acid, excepting for Pro, at the residue 128 retained RpoS activity (Figure 4A), whereas the RpoS activities of variants with hydrophilic amino acids at residue 128 were abolished (Figure 4A). We then conducted a reporter assay with β-galactosidase (Figure 4B). Similarly, the β-galactosidase activities of variants with hydrophilic amino acids at residue 128 were low (Figure 4B). These data indicate that the hydrophobicity of residue 128 is critical for RpoS function.

### Alpha-helix structure near residue 128 is a key factor for RpoS activity

Despite the hydrophobicity of the proline residue, the proline substitution variant displayed no RpoS activity. Proline is generally known as an amino acid that destabilizes secondsary structures of proteins (Gray et al., 1996; Nilsson et al., 1998). The structural analysis for RpoS interacting with RNAP showed that residue 128 is on an α-helix (Liu et al., 2016). Assuming that secondsary structure preservation at residue 128 is another factor that affects RpoS activity, we evaluated the stability of the α-helix in partial structures around residue 128 for the wild-type protein and Asn128 and Pro128 variants using molecular dynamics (MD) simulation (Figure S1). For the calculations, we employed the fragment structure from residues 114 to 135 extracted from the X-ray crystal data of the RpoS-RNAP-4-nt nascent RNA ternary complex (PDB: 5IPL). The time courses of the average root mean squared deviation (RMSD) for the Cα-atoms are shown in Figure S2.

The obtained structures for the wild-type protein and Asn128 mutant indicated α-helicity in this region (Figures S1A,B). Contrarily, mutation of 128 to proline produced a bent structure (Figure S1C); Pro128 was the terminal of an α-helix unit and the secondsary structure from Gly124 to Leu127 was disrupted, and a salt bridge interaction between Arg127 and Glu132 may contribute to the production of such a structure. These data imply that the α-helix structure near residue 128 also plays an important role in RpoS function.

### Residue 128 substitution affects external stress sensitivity

**Figure 5:**
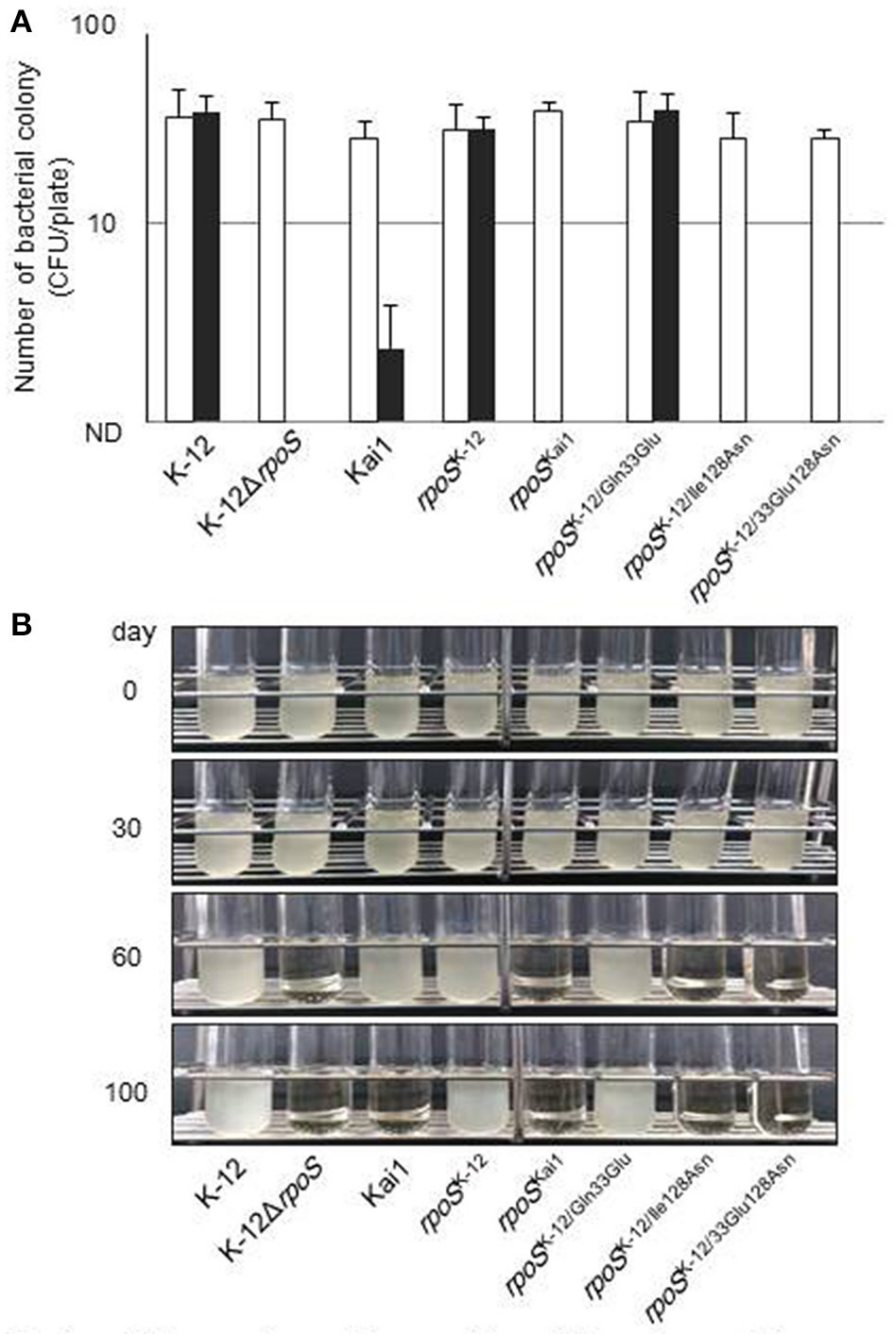
**Effect of substitution of the amino acid at residue 128 on bacterial stress sensitivity. (A)** Bacteria were treated with 20 mM H_2_O_2_ aqueous solution, incubated at 25°C for 2 h, and then plated on LB agar for enumeration of bacterial counts to determine survival after H_2_O_2_ exposure. Filled columns, treated with H_2_O_2_; open columns, untreated control. Mean values are shown (*n* = 3). Error bars represent standard deviations. **(B)** Bacteria were cultured on LB agar at 37°C for 16 h and incubated at 4°C during the study period. Cold-stressed bacteria were cultured in LB broth at 37°C for 24–48 h to determine survival after cold-stress exposure. The experiment was conducted in triplicate, and typical images are shown. The statistical significance of the differences is tabulated in Table S6.

Additionally, we investigated the effect of the substitution at residue 128 on bacterial stress sensitivity (Figure 5). Bacteria expressing various RpoS which encoded by *rpoS*^Kai1^, *rpoS*^K−12^, *rpoS*^K−12/Gln33Glu^, *rpoS*^K−12/Ile128Asn^, or *rpoS*^K−12/Glu33Asn128^ in addition to K−12 and Kai1 strains were exposed to H_2_O_2_ oxidative or cold stresses. Under oxidative stress conditions, *rpoS* variants with the residue 128 substitution (*rpoS*^Kai1^, *rpoS*^K−12/Ile128Asn^, and *rpoS*^K−12/Glu33Asn128^) were more sensitive to oxidative stress than *rpoS*^K−12^; there were no significant differences among the survival rates of *rpoS*^K−12^, *rpoS*^K−12/Gln33Glu^, or strain K−12 (Figure 5A). Of note, strain Kai1 was more resistant to stress than the *rpoS* variants carrying the residue 128 substitution (*rpoS*^Kai1^, *rpoS*^K−12/Ile128Asn^, and *rpoS*^K−12/Glu33Asn128^), implying that strain Kai1 carried known or unknown RpoS-independent stress resistance mechanisms. Similar results were observed under cold stress conditions (Figure 5B); *rpoS* variants with the residue 128 substituted (*rpoS*^*Kai*1^, *rpoS*^K−12/Ile128Asn^, and *rpoS*^K−12/Glu33Asn128^) were more sensitive to cold stress than *rpoS*^K−12^.

### Ile128 or a hydrophobic amino acid at residue 128 is widely conserved among various bacteria

Finally, to investigate importance of Ile128, we searched the *rpoS* sequence of various bacteria in the NCBI gene database (Table 2). *Salmonella enterica* subsp. *enterica* serovar Typhimurium str. LT2, *Pseudomonas aeruginosa* PAO1, *Yersinia pestis* CO92, and *Shigella dysenteriae* Sd197 contain Ile128 (Table 2), indicating that Ile128 is widely conserved in Enterobacteriaceae. We further searched the RpoS sequences of *Legionella pneumophila, Coxiella burnetii*, and *Borrelia burgdorferi* B31, which have Met128, Leu128, and Ile128, respectively (Table 2). Interestingly, *L. pneumophila* and *C. burnetii* have Met and Leu which are hydrophobic; the activity of RpoS with Met128 was equal to that of RpoS with Ile128, and RpoS with Leu128 showed higher activity than that with Ile128 in our study. These data indicate that hydrophobicity of the amino acid at residue 128 is critical for RpoS activity and is consequently important for bacterial survival.

**Table 2:** **Amino acid at residue 128 of RpoS among various bacteria**.

**Strain**	**Amino acid at residue 128**
*Escherichia coli* MG1655	Ile
*Salmonella enterica* subsp. *enterica* serovar Typhimurium str. LT2	Ile
*Pseudomonas aeruginosa* PAO1	Ile
*Yersinia pestis* CO92	Ile
*Shigella dysenteriae* Sd197	Ile
*Legionella pneumophila* subsp. *pneumophila* str. Philadelphia 1	Met
*Coxiella burnetii* RSA 493	Leu
*Borrelia burgdorferi* B31	Ile

## Discussion

RpoS plays an important role in stress resistance (Klauck et al., 2007; Dong and Schellhorn, 2010; Battesti et al., 2011; Bleibtreu et al., 2014; Landini et al., 2014); therefore, many studies have investigated several *rpoS* mutations that modulate RpoS function and its regulation (Hengge-Aronis, 1993; Jishage and Ishihama, 1997; Chi et al., 2009; Dong et al., 2009; Carter et al., 2014; Maharjan and Ferenci, 2015). *rpoS* mutations of laboratory-stocked *E. coli* strains maintained in stab agar, leading to nutritional starvation, were reported to be associated with a high frequency of inactivating alleles (Bleibtreu et al., 2014). In a survey of *rpoS* among 2,040 environmental *E. coli* isolates, RpoS mutants were found to be present in the environment with a frequency of 0.003 among isolates (Chiang et al., 2011). These data indicate that RpoS mutants are generated under low nutrient conditions. The presence of mutant RpoS can be advantageous and disadvantageous: RpoS mutants showed faster growth in the presence of scarce carbon sources but also demonstrated lower stress resistance than strains containing RpoS positive strains (Chiang et al., 2011).

STEC can survive in various potentially stressful environments such as soil, river water, and vegetable surfaces, before infecting humans, and therefore, mutant RpoS is often present in clinically isolated strains. The presence of mutant RpoS may affect infection or pathogenicity of strains: mutant RpoS bearing strains are sensitive to acid and thus may find it difficult to pass the stomach. However, once these mutant RpoS-bearing pathogens reach the gut, they may cause severe damage to the host because wild-type RpoS suppresses STEC virulence factors (Iyoda and Watanabe, 2005; Dong et al., 2009; Dong and Schellhorn, 2010). This may be one reason for the high frequency of mutant RpoS in clinical isolates.

In the present study, we demonstrated that a point mutation in *rpoS* identified in a clinical STEC isolate affected RpoS activity with respect to the transcription of a gene regulated by RpoS; these results indicate that a single point mutation at a hydrophobic residue of the complex formed during transcription leads to a critical change in RpoS regulation.

**Figure 6:**
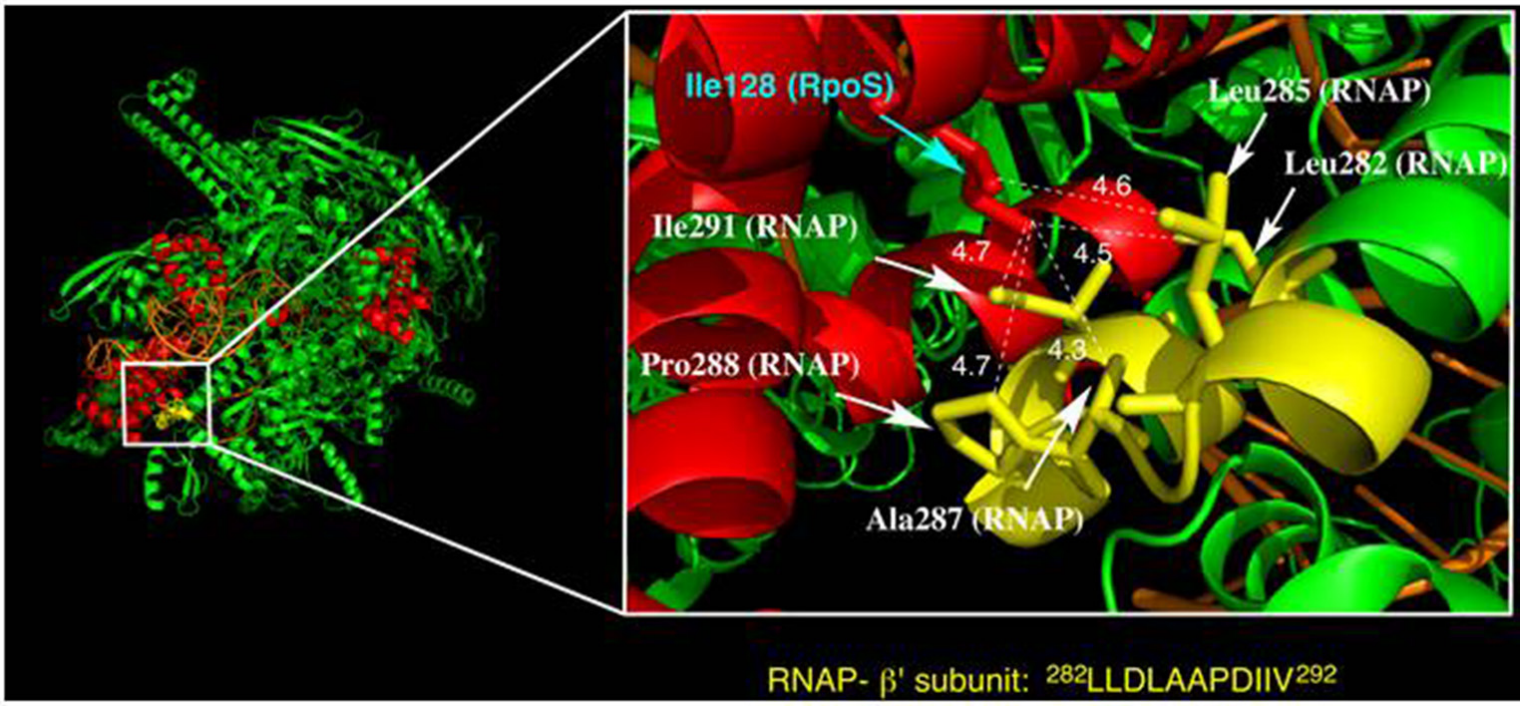
**Structure of a ternary complex of RpoS (red), RNAP (green and yellow), and synthetic oligonucleotide (orange) (from PDB: 5IPL)**. The magnified figure depicts the vicinity of Ile128 in RpoS. Amino acid residues in the RNAP b'-subunit near Ile128 of RpoS are marked in yellow. The moiety marked in yellow is a hydrophobic residue-rich region (see the amino acid sequence). Leu282, Leu285, Ala287, Pro288, and Ile291 in the RNAP b'-subunit face Ile128 of RpoS with distances of <5 Å.

The hydrophobicity of residue 128 was found to be critical for RpoS activity and stress resistance. Notably, the Pro128 variant had no RpoS activity, indicating that α-helicity is also a crucial factor that determines the RpoS regulatory mechanism. According to the X-ray crystal structure of the RpoS-RNAP-4-nt nascent RNA ternary complex (PDB: 5IPL), Ile128 is present in the α-helix of Leu116-Glu132 (Figure 6), which is located at the complex surface. The helix faces two bundle moieties in the RNAP β' subunit (Ser263-Asn309). Several hydrophobic amino acid residues are located near Ile128 (Leu282, Leu285, Ala287, Pro288, and Ile291) with distances of <5 Å, suggesting that wide-range hydrophobic interactions in this area are an important factor in RpoS-RNAP binding. Introduction of a hydrophilic or helix-destroying amino acid residue into position 128 weakens RpoS-RNAP binding because of perturbation in the hydrophobicity of this region, resulting in significant RpoS dysfunction.

The binding ability of the mutant RpoS to RNAP and its detailed underlying mechanism should be assessed in future studies. In addition, a comparison of the crystal structures of mutant and functional RpoS proteins may offer further insight. These experiments can confirm our speculation that the mutation at residue 128 changing a hydrophobic amino acid to a hydrophilic amino acid weakens the hydrophobic interaction between RpoS and RNAP. Additionally, it would be interesting to investigate the effects of amino acid substitutions on RpoS expression because we observed mutations at residue 33 or at residues 33 and 128 to promote RpoS expression (Figure 1, Figure S3).

In conclusion, we found a novel, missense point mutation at RpoS residue 128. This point mutation results in the substitution of a hydrophobic amino acid with a hydrophilic or a helix-destroying amino acid at residue 128, leading to RpoS dysfunction. Remarkably, only one point mutation at a hydrophobic residue of the large macromolecular complex formed in transcription leads to a critical change in RpoS regulation. These findings provide insights on RpoS regulation and further bacterial stress responses.

## Author contributions

TI designed the study. TI, TM, SN carried out experiments. TI, TM, SN, AT, YM discussed in detail about the obtained results. TI and TM wrote a draft, and TI wrote the manuscript.

### Conflict of interest statement

The authors declare that the research was conducted in the absence of any commercial or financial relationships that could be construed as a potential conflict of interest.
