# Supplementary material for: Hydrophobicity of Residue 128 of the Stress-Inducible Sigma Factor RpoS Is Critical for Its Activity
Source: Front Microbiol. 2017 Apr 26;8:656. doi: 10.3389/fmicb.2017.00656 (PMC5405132; doi:10.3389/fmicb.2017.00656)
Supplement: Supplementary file 1 [file DataSheet1.docx]

**Table S1. Statistical significance of results summarized in Figure 2A.**

*P* value

| K-12 vs. K-12Δ*rpoS* | > 0.01 |
| --- | --- |
| K-12 vs. Kai1 | > 0.01 |
| K-12 vs. *rpoS*^K-12^ | > 0.01 |
| K-12 vs. *rpoS*^Kai1^ | > 0.01 |
| K-12 vs. *rpoS*^K-12/Gln33Glu^ | > 0.01 |
| K-12 vs. *rpoS*^K-12/Ile128Asn^ | > 0.01 |
| K-12 vs. *rpoS*^K-12/33Glu128Asn^ | > 0.01 |
| K-12Δ*rpoS* vs. Kai1 | NS |
| K-12Δ*rpoS* vs. *rpoS*^K-12^ | > 0.01 |
| K-12Δ*rpoS* vs. *rpoS*^Kai1^ | NS |
| K-12Δ*rpoS* vs. *rpoS*^K-12/Gln33Glu^ | > 0.01 |
| K-12Δ*rpoS* vs. *rpoS*^K-12/Ile128Asn^ | NS |
| K-12Δ*rpoS* vs. *rpoS*^K-12/33Glu128Asn^ | NS |
| Kai1 vs. *rpoS*^K-12^ | > 0.01 |
| Kai1 vs. *rpoS*^Kai1^ | NS |
| Kai1 vs. *rpoS*^K-12/Gln33Glu^ | > 0.01 |
| Kai1 vs. *rpoS*^K-12/Ile128Asn^ | NS |
| Kai1 vs. *rpoS*^K-12/33Glu128Asn^ | NS |
| *rpoS*^K-12^ vs. *rpoS*^Kai1^ | > 0.01 |
| *rpoS*^K-12^ vs. *rpoS*^K-12/Gln33Glu^ | > 0.01 |
| *rpoS*^K-12^ vs. *rpoS*^K-12/Ile128Asn^ | > 0.01 |
| *rpoS*^K-12^ vs. *rpoS*^K-12/33Glu128Asn^ | > 0.01 |
| *rpoS*^Kai1^ vs. *rpoS*^K-12/Gln33Glu^ | > 0.01 |
| *rpoS*^Kai1^ vs. *rpoS*^K-12/Ile128Asn^ | NS |
| *rpoS*^Kai1^ vs. *rpoS*^K-12/33Glu128Asn^ | NS |
| *rpoS*^K-12/Gln33Glu^ vs. *^rpoS^*^K-12/Gln33Glu^ | > 0.01 |
| *rpoS*^K-12/Gln33Glu^ vs. *^rpoS^*^K-12/33Glu128Asn^ | > 0.01 |
| *rpoS*^K-12/Ile128Asn^ vs. *^rpoS^*^K-12/33Glu128Asn^ | NS |

NS, not significant.

**Table S2. Statistical significance of results summarized in Figure 2B.**

*P* value

| K-12 vs. K-12Δ*rpoS* | > 0.01 |
| --- | --- |
| K-12 vs. Kai1 | > 0.01 |
| K-12 vs. *rpoS*^K-12^ | > 0.05 |
| K-12 vs. *rpoS*^Kai1^ | > 0.01 |
| K-12 vs. *rpoS*^K-12/Gln33Glu^ | NS |
| K-12 vs. *rpoS*^K-12/Ile128Asn^ | > 0.05 |
| K-12 vs. *rpoS*^K-12/33Glu128Asn^ | > 0.05 |
| K-12Δ*rpoS* vs. Kai1 | NS |
| K-12Δ*rpoS* vs. *rpoS*^K-12^ | > 0.01 |
| K-12Δ*rpoS* vs. *rpoS*^Kai1^ | NS |
| K-12Δ*rpoS* vs. *rpoS*^K-12/Gln33Glu^ | > 0.01 |
| K-12Δ*rpoS* vs. *rpoS*^K-12/Ile128Asn^ | NS |
| K-12Δ*rpoS* vs. *rpoS*^K-12/33Glu128Asn^ | NS |
| Kai1 vs. *rpoS*^K-12^ | > 0.01 |
| Kai1 vs. *rpoS*^Kai1^ | NS |
| Kai1 vs. *rpoS*^K-12/Gln33Glu^ | > 0.01 |
| Kai1 vs. *rpoS*^K-12/Ile128Asn^ | NS |
| Kai1 vs. *rpoS*^K-12/33Glu128Asn^ | NS |
| *rpoS*^K-12^ vs. *rpoS*^Kai1^ | > 0.01 |
| *rpoS*^K-12^ vs. *rpoS*^K-12/Gln33Glu^ | NS |
| *rpoS*^K-12^ vs. *rpoS*^K-12/Ile128Asn^ | > 0.01 |
| *rpoS*^K-12^ vs. *rpoS*^K-12/33Glu128Asn^ | > 0.01 |
| *rpoS*^Kai1^ vs. *rpoS*^K-12/Gln33Glu^ | > 0.01 |
| *rpoS*^Kai1^ vs. *rpoS*^K-12/Ile128Asn^ | NS |
| *rpoS*^Kai1^ vs. *rpoS*^K-12/33Glu128Asn^ | NS |
| *rpoS*^K-12/Gln33Glu^ vs. *rpoS*^K-12/Ile128Asn^ | > 0.01 |
| *rpoS*^K-12/Gln33Glu^ vs. *rpoS*^K-12/33Glu128Asn^ | > 0.01 |
| *rpoS*^K-12/Ile128Asn^ vs. *rpoS*^K-12/33Glu128Asn^ | NS |

NS, not significant.

**Table S3. Statistical significance of results summarized in Figure 3A.**

*P* value

| *rpoS*^K-12^ vs. *rpoS*^Kai1^ | > 0.01 |
| --- | --- |
| *rpoS*^K-12^ vs. *rpoS*^K-12/Gln33Glu^ | > 0.01 |
| *rpoS*^K-12^ vs. *rpoS*^K-12/Ile128Asn^ | > 0.01 |
| *rpoS*^K-12^ vs. *rpoS*^K-12/33Glu128Asn^ | > 0.01 |
| *rpoS*^Kai1^ vs. *rpoS*^K-12/Gln33Glu^ | > 0.01 |
| *rpoS*^Kai1^ vs. *rpoS*^K-12/Ile128Asn^ | NS |
| *rpoS*^Kai1^ vs. *rpoS*^K-12/33Glu128Asn^ | NS |
| *rpoS*^K-12/Gln33Glu^ vs. *rpoS*^K-12/Ile128Asn^ | > 0.01 |
| *rpoS*^K-12/Gln33Glu^ vs. *rpoS*^K-12/33Glu128Asn^ | > 0.01 |
| *rpoS*^K-12/Ile128Asn^ vs. *rpoS*^K-12/33Glu128Asn^ | NS |

NS, not significant.

**Table S4. Statistical significance of results summarized in Figure 4A.**

*P* value

| Ile vs. Ala | > 0.01 |
| --- | --- |
| Ile vs. Leu | > 0.01 |
| Ile vs. Met | NS |
| Ile vs. Phe | > 0.01 |
| Ile vs. Pro | > 0.01 |
| Ile vs. Arg | > 0.01 |
| Ile vs. Asp | > 0.01 |
| Ile vs. Asn | > 0.01 |
| Ile vs. Gln | > 0.01 |
| Ile vs. Glu | > 0.01 |
| Ile vs. Lys | > 0.01 |
| Ala vs. Leu | > 0.01 |
| Ala vs. Met | > 0.01 |
| Ala vs. Phe | NS |
| Ala vs. Pro | > 0.01 |
| Ala vs. Arg | > 0.01 |
| Ala vs. Asp | > 0.01 |
| Ala vs. Asn | > 0.01 |
| Ala vs. Gln | > 0.01 |
| Ala vs. Gln | > 0.01 |
| Ala vs. Lys | > 0.01 |
| Leu vs. Met | > 0.01 |
| Leu vs. Phe | > 0.01 |
| Leu vs. Pro | > 0.01 |
| Leu vs. Arg | > 0.01 |
| Leu vs. Asp | > 0.01 |
| Leu vs. Asn | > 0.01 |
| Leu vs. Gln | > 0.01 |
| Leu vs. Gln | > 0.01 |
| Leu vs. Lys | > 0.01 |

**Table S4 (continued).**

| Met vs. Phe | > 0.01 |
| --- | --- |
| Met vs. Pro | > 0.01 |
| Met vs. Arg | > 0.01 |
| Met vs. Asp | > 0.01 |
| Met vs. Asn | > 0.01 |
| Met vs. Gln | > 0.01 |
| Met vs. Gln | > 0.01 |
| Met vs. Lys | > 0.01 |
| Phe vs. Pro | > 0.01 |
| Phe vs. Arg | > 0.01 |
| Phe vs. Asp | > 0.01 |
| Phe vs. Asn | > 0.01 |
| Phe vs. Gln | > 0.01 |
| Phe vs. Gln | > 0.01 |
| Phe vs. Lys | > 0.01 |
| Pro vs. Arg | NS |
| Pro vs. Asp | NS |
| Pro vs. Asn | NS |
| Pro vs. Gln | NS |
| Pro vs. Gln | NS |
| Pro vs. Lys | NS |
| Arg vs. Asp | NS |
| Arg vs. Asn | NS |
| Arg vs. Gln | NS |
| Arg vs. Gln | NS |
| Arg vs. Lys | NS |
| Asp vs. Asn | NS |
| Asp vs. Gln | NS |
| Asp vs. Gln | NS |
| Asp vs. Lys | NS |
| Asn vs. Gln | NS |

NS, not significant.

**Table S5. Statistical significance of results summarized in Figure 4B.**

*P* value

| Ile vs. Ala | > 0.01 |
| --- | --- |
| Ile vs. Leu | NS |
| Ile vs. Met | NS |
| Ile vs. Phe | > 0.01 |
| Ile vs. Pro | > 0.01 |
| Ile vs. Arg | > 0.01 |
| Ile vs. Asp | > 0.01 |
| Ile vs. Gln | > 0.01 |
| Ile vs. Glu | > 0.01 |
| Ile vs. Lys | > 0.01 |
| Ala vs. Leu | > 0.01 |
| Ala vs. Met | > 0.01 |
| Ala vs. Phe | > 0.01 |
| Ala vs. Pro | > 0.01 |
| Ala vs. Arg | > 0.01 |
| Ala vs. Asp | > 0.01 |
| Ala vs. Gln | > 0.05 |
| Ala vs. Glu | > 0.01 |
| Ala vs. Lys | > 0.01 |
| Leu vs. Met | NS |
| Leu vs. Phe | > 0.01 |
| Leu vs. Pro | > 0.01 |
| Leu vs. Arg | > 0.01 |
| Leu vs. Asp | > 0.01 |
| Leu vs. Gln | > 0.01 |
| Leu vs. Glu | > 0.01 |
| Leu vs. Lys | > 0.01 |

**Table S5 (continued).**

| Met vs. Phe | > 0.01 |
| --- | --- |
| Met vs. Pro | > 0.01 |
| Met vs. Arg | > 0.01 |
| Met vs. Asp | > 0.01 |
| Met vs. Gln | > 0.01 |
| Met vs. Glu | > 0.01 |
| Met vs. Lys | > 0.01 |
| Phe vs. Pro | > 0.01 |
| Phe vs. Arg | > 0.01 |
| Phe vs. Asp | > 0.01 |
| Phe vs. Gln | > 0.01 |
| Phe vs. Glu | > 0.01 |
| Phe vs. Lys | > 0.01 |
| Pro vs. Arg | NS |
| Pro vs. Asp | NS |
| Pro vs. Gln | NS |
| Pro vs. Glu | NS |
| Pro vs. Lys | NS |
| Arg vs. Asp | NS |
| Arg vs. Gln | NS |
| Arg vs. Glu | NS |
| Arg vs. Lys | NS |
| Asp vs. Gln | NS |
| Asp vs. Glu | NS |
| Asp vs. Lys | NS |
| Gln vs. Glu | NS |
| Gln vs. Lys | NS |
| Glu vs. Lys | NS |

NS, not significant.

**Table S6. Statistical significance of results summarized in Figure 5A.**

*P* value

| K-12 vs. K-12Δ*rpoS* | > 0.01 |
| --- | --- |
| K-12 vs. Kai1 | > 0.01 |
| K-12 vs. *rpoS*^K-12^ | NS |
| K-12 vs. *rpoS*^Kai1^ | > 0.01 |
| K-12 vs. *rpoS*^K-12/Gln33Glu^ | NS |
| K-12 vs. *rpoS*^K-12/Ile128Asn^ | > 0.01 |
| K-12 vs. *rpoS*^K-12/33Glu128Asn^ | > 0.01 |
| K-12Δ*rpoS* vs. Kai1 | > 0.01 |
| K-12Δ*rpoS* vs. *rpoS*^K-12^ | > 0.01 |
| K-12Δ*rpoS* vs. *rpoS*^Kai1^ | NS |
| K-12Δ*rpoS* vs. *rpoS*^K-12/Gln33Glu^ | > 0.01 |
| K-12Δ*rpoS* vs. *rpoS*^K-12/Ile128Asn^ | NS |
| K-12Δ*rpoS* vs. *rpoS*^K-12/33Glu128Asn^ | NS |
| Kai1 vs. *rpoS*^K-12^ | > 0.01 |
| Kai1 vs. *rpoS*^Kai1^ | NS |
| Kai1 vs. *rpoS*^K-12/Gln33Glu^ | > 0.01 |
| Kai1 vs. *rpoS*^K-12/Ile128Asn^ | NS |
| Kai1 vs. *rpoS*^K-12/33Glu128Asn^ | NS |
| *rpoS*^K-12^ vs. *rpoS*^Kai1^ | > 0.01 |
| *rpoS*^K-12^ vs. *rpoS*^K-12/Gln33Glu^ | NS |
| *rpoS*^K-12^ vs. *rpoS*^K-12/Ile128Asn^ | > 0.01 |
| *rpoS*^K-12^ vs. *rpoS*^K-12/33Glu128Asn^ | > 0.01 |
| *rpoS*^Kai1^ vs. *rpoS*^K-12/Gln33Glu^ | > 0.01 |
| *rpoS*^Kai1^ vs. *rpoS*^K-12/Ile128Asn^ | NS |
| *rpoS*^Kai1^ vs. *rpoS*^K-12/33Glu128Asn^ | NS |
| *rpoS*^K-12/Gln33Glu^ vs. *rpoS*^K-12/Ile128Asn^ | > 0.01 |
| *rpoS*^K-12/Gln33Glu^ vs. *rpoS*^K-12/33Glu128Asn^ | > 0.01 |
| *rpoS*^K-12/Ile128Asn^ vs. *rpoS*^K-12/33Glu128Asn^ | NS |

NS, not significant.

**Table S7. Strains and plasmids used in this study.**

　　　　Strain 　 Description

| *E. coli* strain Kai1 | A clinical Shiga toxin-producing *E. coli* isolate |
| --- | --- |
| *E. coli* strain K-12 BW25113 | A laboratory strain |
| K-12∆*rpoS* | Keio clone which deleted *rpoS*, distributed by National Institute for Genetics, Shizuoka, Japan |
| K-12∆*rpoS* with *rpoS*^K-12^ | K-12∆*rpoS* with pSTV28 harboring K-12 *rpoS* and its promoter |
| K-12∆*rpoS* with *rpoS*^Kai1^ | K-12∆rpoS with pSTV28 harboring Kai1 *rpoS* and its promoter |
| K-12∆*rpoS* with *rpoS*^K-12/Gln33Glu^ | K-12∆rpoS with pSTV28 harboring *rpoS*^K-12/Gln33Glu^ |
| K-12∆*rpoS* with *rpoS*^K-12/Ile128Asn^ | K-12∆rpoS with pSTV28 harboring *rpoS*^K-12/Ile128Asn^ |
| K-12∆*rpoS* with *rpoS*^K-12/Glu33Asn128^ | K-12∆rpoS with pSTV28 harboring *rpoS*^K-12/Glu33Asn128^ |
| K-12∆*rpoS* with *rpoS*^K-12/Ile128Ala^ | K-12∆rpoS with pSTV28 harboring *rpoS*^K-12/Ile128Ala^ |
| K-12∆*rpoS* with *rpoS*^K-12/Ile128Arg^ | K-12∆rpoS with pSTV28 harboring *rpoS*^K-12/Ile128Arg^ |
| K-12∆*rpoS* with *rpoS*^K-12/Ile128Asp^ | K-12∆rpoS with pSTV28 harboring *rpoS*^K-12/Ile128Asp^ |
| K-12∆*rpoS* with *rpoS*^K-12/Ile128Gln^ | K-12∆rpoS with pSTV28 harboring *rpoS*^K-12/Ile128Gln^ |
| K-12∆*rpoS* with *rpoS*^K-12/Ile128Glu^ | K-12∆rpoS with pSTV28 harboring *rpoS*^K-12/Ile128Glu^ |
| K-12∆*rpoS* with *rpoS*^K-12/Ile128Leu^ | K-12∆rpoS with pSTV28 harboring *rpoS*^K-12/Ile128Leu^ |
| K-12∆*rpoS* with *rpoS*^K-12/Ile128Lys^ | K-12∆rpoS with pSTV28 harboring *rpoS*^K-12/Ile128Lys^ |
| K-12∆*rpoS* with *rpoS*^K-12/Ile128Met^ | K-12∆rpoS with pSTV28 harboring *rpoS*^K-12/Ile128Met^ |
| K-12∆*rpoS* with *rpoS*^K-12/Ile128Phe^ | K-12∆rpoS with pSTV28 harboring *rpoS*^K-12/Ile128Phe^ |
| K-12∆*rpoS* with *rpoS*^K-12/Ile128Pro^ | K-12∆rpoS with pSTV28 harboring *rpoS*^K-12/Ile128Pro^ |
| RLG10438 | Parental strain VH1000 is *E. coli* MG1655 with a *lacZ* deletion. All β-galactosidase activity originates from *osmY* promoter-*lacZ* fusion, single copy on the chromosome in a lambda prophage at the att site (45). |
| RLG10438 with *rpoS*^K-12^ | RLG10438 with pSTV28 harboring K-12 *rpoS* and its promoter |
| RLG10438 with *rpoS*^Kai1^ | RLG10438 with pSTV28 harboring Kai1 *rpoS* and its promoter |
| RLG10438 with *rpoS*^K-12/Gln33Glu^ | RLG10438 with pSTV28 harboring *rpoS*^K-12/Gln33Glu^ |
| RLG10438 with *rpoS*^K-12/Ile128Asn^ | RLG10438 with pSTV28 harboring *rpoS*^K-12/Ile128Asn^ |
| RLG10438 with *rpoS*^K-12/Glu33Asn128^ | RLG10438 with pSTV28 harboring *rpoS*^K-12/Glu33Asn128^ |
| *E. coli* K-12 DH5α | A host for genetic manipulation and plasmid maintain |
| pSTV28 | A low-copy-number plasmid with the chloramphenicol-resistant gene (Takara, Japan) and with 15A origin derived from p15A |

**Table S8. Primers for amino acid substitution at residue 33 or 128 of K-12 RpoS.**

　 Oligonucleotide 　Sequence

| to Gln at residue 33 to Glu_F  to Gln at residue 33 to Glu_R | 5′-AGTAGAAGAGGAACCCAGTGATAACGA-3′  5′-GGTTCCTCTTCTACTAAGGCCTTTTCG-3′ |
| --- | --- |
| to Ile at residue 128 to Ala_F  to Ile at residue 128 to Ala_R | 5′-TGGCCCGCGCGGTAGAGAAGTTTGACC-3′  5′-TACCGCGCGGGCCAGCCCCAG-3′ |
| to Ile at residue 128 to Arg_F  to Ile at residue 128 to Arg_R | 5′-TGCGTCGCGCGGTAGAGAAGTTTGACC-3′  5′-TACCGCGCGACGCAGCCCCAG-3′ |
| to Ile at residue 128 to Asp_F  to Ile at residue 128 to Asp_R | 5′-TGCGCCGCGCGGTAGAGAAGTTTGACC-3′  5′-TACCGCGCGGCGCAGCCCCAG-3′ |
| to Ile at residue 128 to Gln_F  to Ile at residue 128 to Glu_R | 5′-TGCAACGCGCGGTAGAGAAGTTTGACC-3′  5′-TACCGCGCGTTGCAGCCCCAG-3′ |
| to Ile at residue 128 to Gln_F  to Ile at residue 128 to Gln_R | 5′-TGGAACGCGCGGTAGAGAAGTTTGACC-3′  5′-TACCGCGCGTTCCAGCCCCAG-3′ |
| to Ile at residue 128 to Leu_F  to Ile at residue 128 to Leu_R | 5′-TGCTCCGCGCGGTAGAGAAGTTTGACC-3′  5′-TACCGCGCGGAGCAGCCCCAG-3′ |
| to Ile at residue 128 to Lys_F  to Ile at residue 128 to Lys_R | 5′-TGAAGCGCGCGGTAGAGAAGTTTGACC-3′  5′-TACCGCGCGCTTCAGCCCCAG-3′ |
| to Ile at residue 128 to Met_F  to Ile at residue 128 to Met_R | 5′-TGATGCGCGCGGTAGAGAAGTTTGACC-3′  5′-TACCGCGCGCATCAGCCCCAG-3′ |
| to Ile at residue 128 to Phe_F  to Ile at residue 128 to Pne_R | 5′-TGTTCCGCGCGGTAGAGAAGTTTGACC-3′  5′-TACCGCGCGGAACAGCCCCAG-3′ |
| to Ile at residue 128 to Pro_F  to Ile at residue 128 to Pro_R | 5′-TGCCACGCGCGGTAGAGAAGTTTGACC-3′  5′-TACCGCGCGTGGCAGCCCCAG-3′ |

**Table S9. Primers used in qRT-PCR.**

　 Oligonucleotide 　Sequence

| katE_F  katE_R | 5′-CCGAAAACGAACAAGCAG-3′  5′-CCAGACCAGGAACAATATGAC-3′ |
| --- | --- |
| glgS_F  glgS_R | 5′-GTTTTGCCAGAATGCACG-3′  5′-CAGTAACGGCCAGAATATCG-3′ |
| rpoS_F  rpoS_R | 5′-GCTTCTCAACATACGCAACC-3′  5′-CATAATCGCCCGTTCAATC-3′ |
| rrsA_F  rrsA_R | 5′-CGTCGCAAGACCAAAGAG-3′  5′-CTAATCCCATCTGGGCAC-3′ |

**Fig. S1. Molecular dynamics (MD) calculated structures for fragment Leu114-Asp135 in RpoS.**

(A) Wild-type protein (Ile128); (B) Ile128Asn variant; (C) Ile128Pro variant. The calculations were conducted using AMBER 03 force field, with point charges at pH=7.4 and an aqueous solution model of 0.9% NaCl aq with additional Na^+^ and Cl^-^ for neutralization at 310.15 K. Hydrogen atoms and water molecules are not depicted for simplicity. Amino acid sequence: ^114^LALLDLIEEGNLGL^128^IRAVEKFD^135^.

**Fig. S2. Time course of RMSD (root mean standard deviation) of Cα atoms during molecular dynamics (MD) calculations for fragment Leu114-Asp135 in RpoS.**

(A) wild-type (Ile128) protein; (B) Ile128Asn variant; (C) Ile128Pro variant. The calculations were conducted using AMBER 03 force field with point charges at pH=7.4 and an aqueous solution model of 0.9% NaCl aq with additional Na^+^ and Cl^-^ for neutralization at 310.15 K.

**Fig. S3. Effect of mutations on RpoS expression.**

Effects of mutations on *rpoS* and *rpoS*-regulated gene expression and on RpoS expression were investigated. (A) mRNA expression in bacteria harvested at O/N culture (16 hr) was analyzed using quantitative reverse transcriptional PCR (qRT-PCR) with gene-specific primers and SuperScript III Platinum One-Step qRT-PCR Kit (Invitrogen), according to manufacturer instructions. *rrsA* encoding 16S rRNA was used as a reference gene for normalization of qRT-PCR. Primers used in this experiment are shown in Table S9. (B) Densitometry analysis of the RpoS expression for the data presented in Figure 1 was performed using imaging software, ImageQuant TL (GE Healthcare).
