# Supplementary figures and images for: Hydrophobicity of Residue 128 of the Stress-Inducible Sigma Factor RpoS Is Critical for Its Activity
Source: Front Microbiol. 2017 Apr 26;8:656. doi: 10.3389/fmicb.2017.00656 (PMC5405132; doi:10.3389/fmicb.2017.00656)

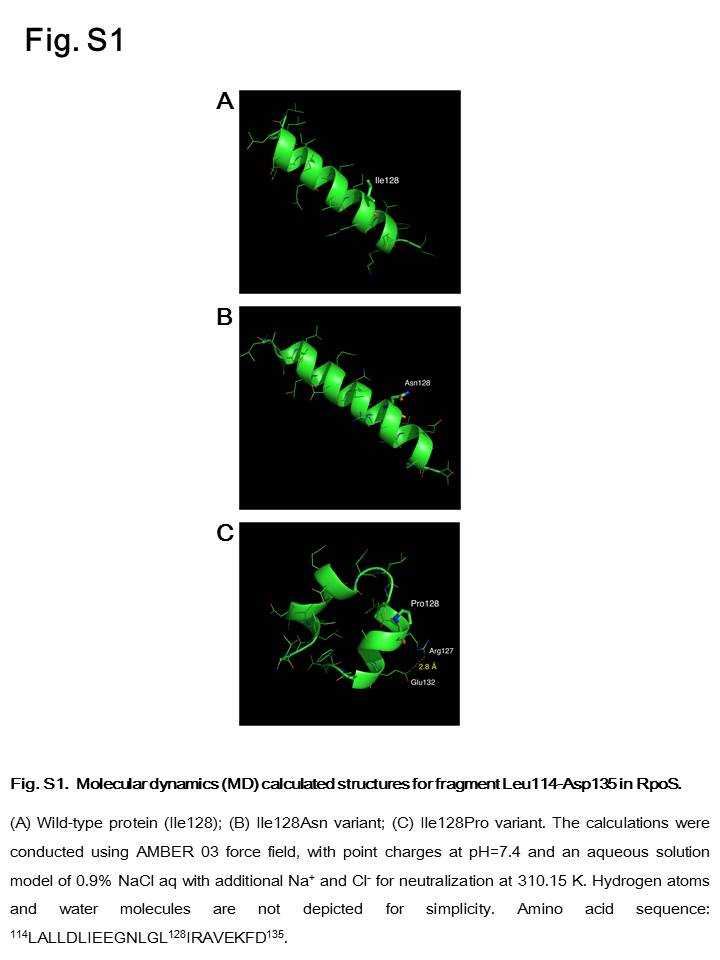

Supplement: Supplementary file 2 [file Image1.JPEG]

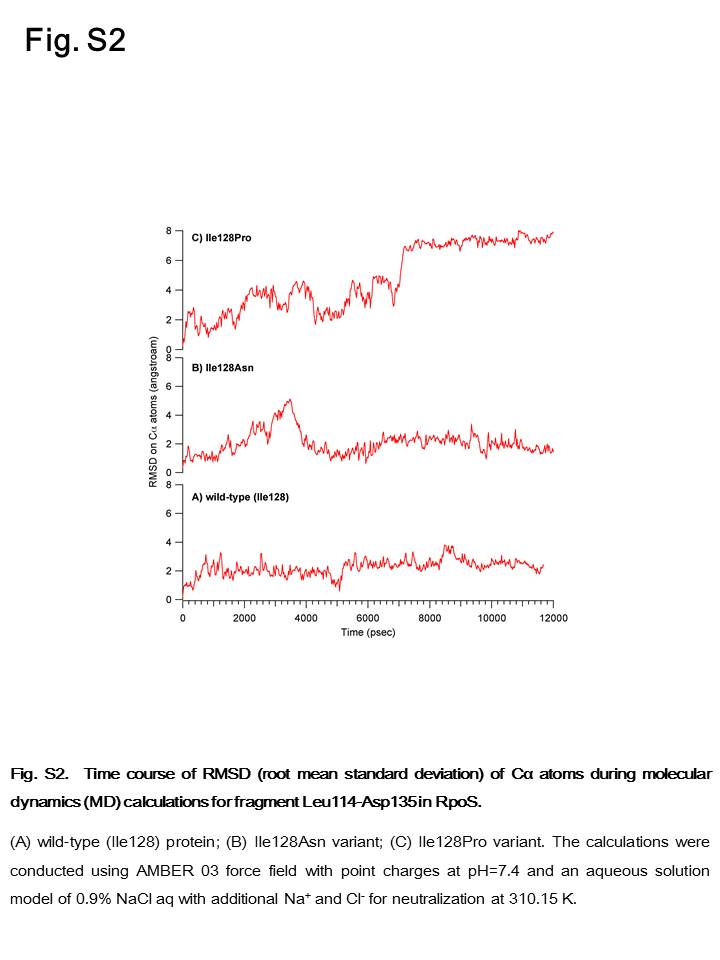

Supplement: Supplementary file 3 [file Image2.JPEG]

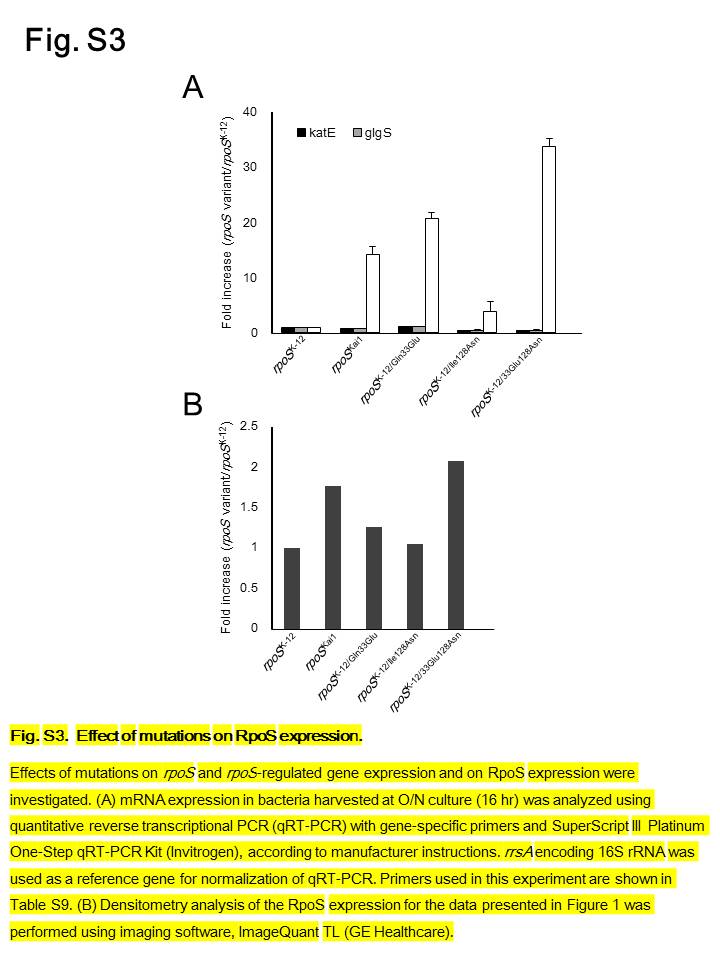

Supplement: Supplementary file 4 [file Image3.JPEG]
